# Excessive Activation of TGFβ by Spinal Instability Causes Vertebral Endplate Sclerosis

**DOI:** 10.1038/srep27093

**Published:** 2016-06-03

**Authors:** Qin Bian, Amit Jain, Xin Xu, Khaled Kebaish, Janet L. Crane, Zhendong Zhang, Mei Wan, Lei Ma, Lee H. Riley, Paul D. Sponseller, X. Edward Guo, Willian Weijia Lu, Yongjun Wang, Xu Cao

**Affiliations:** 1 Department of Orthopaedic Surgery, Institute for Cell Engineering, Johns Hopkins University, Baltimore, MD 21205, USA; 2 Institute of Spine, Longhua Hospital, Shanghai University of Traditional Chinese Medicine, Shanghai, 200032, P. R. China; 3 State Key Laboratory of Oral Disease, West China School of Stomatology, Sichuan University, Chengdu, Sichuan, 610041, P. R. China; 4 Department of Pediatrics, Johns Hopkins University, Baltimore, MD 21287, USA; 5 Bone Bioengineering Laboratory, Department of Biomedical Engineering, Columbia University, New York, NY 10027, USA; 6 Department of Orthopaedics and Traumatology, Li Ka Shing Faculty of Medicine, The University of Hong Kong, Pokfulam, China

## Abstract

Narrowed intervertebral disc (IVD) space is a characteristic of IVD degeneration. EP sclerosis is associated with IVD, however the pathogenesis of EP hypertrophy is poorly understood. Here, we employed two spine instability mouse models to investigate temporal and spatial EP changes associated with IVD volume, considering them as a functional unit. We found that aberrant mechanical loading leads to accelerated ossification and hypertrophy of EP, decreased IVD volume and increased activation of TGFβ. Overexpression of active TGFβ in CED mice showed a similar phenotype of spine instability model. Administration of TGFβ Receptor I inhibitor attenuates pathologic changes of EP and prevents IVD narrowing. The aberrant activation of TGFβ resulting in EPs hypertrophy-induced IVD space narrowing provides a pharmacologic target that could have therapeutic potential to delay DDD.

Degenerative disc disease (DDD) is one of the most common musculoskeletal disorders that is associated with disability and absenteeism from work. Degeneration has been detected as early as teenage years and severe degeneration is found in 60% of 70-year olds^1^,^2^. DDD typically presents with back pain and imposes an enormous socio-economic burden, over $100 billion annually in the US alone. This cost exceeds the combined costs of stroke, respiratory infection, diabetes, coronary artery disease and rheumatoid disease^1^,^2^. Several factors have been implicated to cause DDD such as aging, genetic predisposition, toxic factors, metabolic disorders, low-grade infection, neurogenic inflammation and mechanical factors^3^. However, the pathogenesis of DDD under mechanical loading environment is not well known.

Intervertebral disc (IVD) is composed of three parts: gel-like nucleus pulposus (NP) in the central compartment surrounded by an annulus fibrosis (AF) ring, and both cranial and caudal cartilage endplates (EPs) that attach the IVD inner ring to the adjacent vertebrae. EPs transmit mechanical loads produced by body weight and muscle activity between the bony vertebrae and soft tissue^4^. Moreover, EPs serve as a selective permeability barrier, allowing passage of small solutes, such as nutrient substances but impeding transport of larger solutes such as inflammatory factors^5^. Sclerosis of EPs change the mechanical property and impair diffusion and nutrient supply, thus accelerating IVD degeneration^5^. However, the originating mechanism of EP pathology is still not clearly understood.

EPs undergo calcification and ossification and become sclerotic with aging^6^. We have previously found that excess activation of TGFβ causes sclerosis and angiogenesis of subchondral bone in the knee joint, which alters loading distribution on articular cartilage and is key in the pathogenesis of osteoarthritis (OA)^7^,^8^. Upregulation of TGFβ has been observed in calcified hypertrophic EPs of degenerative IVD^9^. Whether TGFβ is involved in EP sclerotic changes is unknown.

In this study, we investigated temporal and spatial EP changes resulting from mechanical stress by focusing on two spine instability mouse models. We found that destabilization of the spine resulted in accelerated ossification and increased volume of EP, decreased IVD volume, and increased levels of active TGFβ. Transgenic expression of active TGFβ in CED mice showed similar results to the spine instability model, whereas administration of TGFβ Receptor I inhibitor attenuated EP and IVD volume changes. Our findings suggest that inhibition of TGFβ targeting EP degeneration may be a potential therapeutic target for DDD.

## Materials and Methods

### Animal models

Lumbar Spine instability mouse model: C57BL/6J (male, 8-week-old, Charles River) mice were operated by resection of the lumbar 3^th^ - Lumbar 5^th^ (L_3_–L_5_) spinous processes along with the supraspinous and interspinous ligaments to induce instability of lumbar spine^10^,^11^. Sham operations were done only by detachment of the posterior paravertebral muscles from the L_3_–L_5_ vertebrae. The operated mice were intraperitoneally injected with either TβRI inhibitor (1 mg/kg, SB-505124, Sigma-Aldrich) (SB group) or the equivalent volume of vehicle (DMSO) (Sham and Veh group) once every two days. Mice (8-week old) were euthanized at 2, 4, and 8 w after the surgery (*n* = 8 per group).

Caudal spine instability mouse model (CSI): the instability of caudal spine was induced by fully depth annular stab and nucleus pulposus (NP) removal of the caudal 7^th^–8^th^ (C_7–8_) IVD^12^. C_7–8_ EPs were observed at 4 w post-surgery (*n* = 6 per group). The treatments were the same as those described in the first model.

CED mouse: CED mice were generated in our laboratory as previously described, in which cells driven by a 2.3-kb type I collagen promoter specifically expressed CED-derived TGF-β1 mutation (H222D)^13^. Six months old mice were observed (*n* = 6 per group).

*Nestin-Cre*^*TM*^*Er::Rosa26-lacZ*^*flox/flox*^ mouse: This mouse was generated in our laboratory as prevously described^7^. *Nestin-Cre*^*TM*^*Er*mice were crossed with *(ROSA)26Sortm1Sor/J* mice to obtain *Nestin-Cre*^*TM*^*Er::Rosa26-lacZ*^*flox/flox*^offspring. We performed sham or LSI operation on 8 weeks old male *Nestin-Cre*^*TM*^*Er::Rosa26-lacZ*^*flox/flox*^ mice and their WT littermates. After the surgery, each group was treated with 100 mg/kg body weight of tamoxifen daily for 4 weeks (n = 3 per group).

All experimental protocols were reviewed and approved by the Institutional Animal Care and Use Committee of the Johns Hopkins University, Baltimore, MD, USA, and carried out in accordance with the approved guidelines.

### μCT

The lower thoracic and whole lumbar spine from mice were dissected, fixed in 10% buffered formalin for 48 h and then transferred into PBS, examined by high-resolution μCT (Skyscan1172). The ribs on the lower thoracic were included for identification of L_4_–L_5_ IVD localization. Images were reconstructed and analyzed using NRecon v1.6 and CTAn v1.9, respectively. Three-dimensional model visualization software, CTVol v2.0, was used to analyze parameters of the L_4_–L_5_ IVD with half height of L_4_ and L_5_ vertebrae. The scanner was set at a voltage of 49 kVp, a current of 200 μA and a resolution of 6.8 μm per pixel to measure the IVD and EP. A resolution 16.8 μm of per pixel was set for the whole L_5_ vertebral body measurement. Coronal images of the L_4_–L_5_ IVD were used to perform three-dimensional histomorphometric analyses of IVD and cartilage EP while sagittal images of L_5_ vertebra were used for those of vertebral body. IVD volume was defined by the region of interest (ROI) to cover the whole invisible space between L_4_–L_5_ vertebrae. Cartilage EP volume was defined to cover visible bony plate close to the vertebrae. L_5_ vertebral body TV was described to figure out the medial compartment excluding cortical bone, transverse and spinous processes. A total of five consecutive images of ROI were used for showing three-dimensional reconstruction of IVD space and EP. Three-dimensional structural parameters analyzed included: TV (total tissue volume), and Trabecular separation distribution.

### CT-based microangiography

Blood vessels in EP sites were imaged by angiography of microphil-perfused bones. In detail, the thoracic cavity of mice was opened after anesthesia, and the inferior vena cava was severed. The vascular system was flushed with PBS containing heparin sodium (100 U/ml) through a needle inserted into the left ventricle. The specimens were then pressure fixed with 10% neutral buffered formalin which was washed off from the vessels by heparinized saline solution. A radio paque silicone rubber compound containing lead chromate (Microfil MV-122, Flow Tech) was injected to label the vasculature. Samples were stored at 4 °C overnight for contrast agent polymerization. Mouse C_8–9_ along with EPs were dissected and decalcified in 10% EDTA after fixation in 10% neutral buffered formalin for 24 h. Images were obtained using a μCT imaging system (Skyscan 1172).

### Histochemistry, immunohistochemistry and histomorphometry

The specimens were fixed in 10% buffered formalin for 48 h, decalcified in 10% EDTA (pH 7.4) for 14 d, dehydrated and embedded in paraffin. Four-micrometer-thick coronal-oriented sections of the L_4_–L_5_, or C_8–10_ spine were processed for safranin O and fast green staining. Tartrate-resistant acid phosphatase (Trap) staining was performed using a standard protocol (Sigma-Aldrich). Immunostaining was performed using a standard protocol. Sections were incubated with primary antibodies to mouse Collagen II (Abcam, 1:200, ab34712), osterix (Abcam, 1:600, ab22552), osteocalcin (Takara Bio Inc., 1:200, M137), nestin (Aves Labs, Inc., 1:300, NES0407), CD31 (Abcam, 1:100, ab28364), Collagen I (Santa Cruz, 1:100, sc-25974), Collagen X (Abcam, 1:200, ab58632), cathepsin K (Santa Cruz, 1:100, sc-48353), pSmad2/3 (Santa Cruz, 1:200, sc-11769) at 4 °C overnight. For immunohistochemical staining, a horse radish peroxidase–streptavidin detection system (Dako) was subsequently used to detect the immunoactivity, followed by counterstaining with hematoxylin (Sigma-Aldrich). For immunofluorescent assay, the slides were incubated with secondary antibodies conjugated with fluorescence at room temperature for 1 h while avoiding light. Morphometric study was performed by an image auto-analysis system (Olympus DP71). Quantitative histomorphometric analysis was conducted in a blinded fashion with Image-Pro Plus Software version 6.0 (Media Cybernetics Inc). EP score were obtained as previously described^1^,^14^. The osterix or osteocalcin positive cells was obtained by counting the number of positive staining cells in the holes of EP region. The area of CD31 or nestin positive staining was calculated in the holes of EP region. The area of Col I, X and Cathepsin K positive staining was calculated in the whole EP region.

### ELISA

The concentration of total TGFβ1 and active TGFβ1 was determined in the L_1–3_ IVDs using the ELISA Development kit (R&D Systems) according to the manufacturer’s instructions (n = 3 per group).

### Statistics

The data was expressed as mean ± s.d., and statistical significance was determined using a Student *t* test in time-point or genetic mice comparison, or One-Way ANOVA followed by a post-hoc LSD test (homogeneity of variance) or a Tukey’s test (heterogeneity of variance) in treatment comparison. The level of significance was defined as *p* < 0.05. All data analyses were performed using SPSS 15.0 analysis software (SPSS Inc).

## Results

### Aberrant mechanical loading in the spine leads to narrowing of IVD space

**Figure 1:**
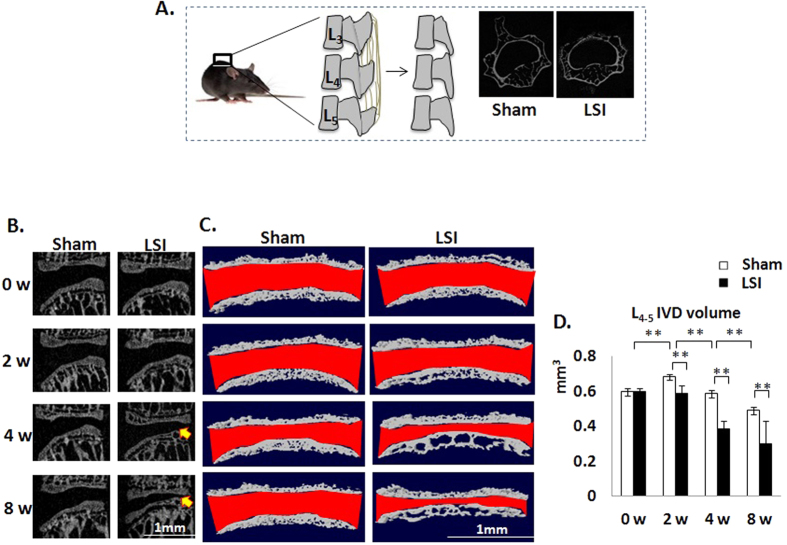
IVD space is narrowed in LSI mice. (**A**) Lumbar spine instability mouse model (LSI). Mouse L_3_–L_5_spinous processes were resected along with the supraspinous and interspinous ligaments to induce instability of lumbar spine. (**B**) Mid-sagittalL_4_–L_5_μCT image of LSI and sham mice. Arrows indicate narrowed IVD space. (**C**) Coronal plane of cranial and caudal L_4_–L_5_EPs (3D reconstruction of μCT image). IVD space indicated by red color. (**D**) Quantification of L_4_–L_5_ IVD volume. Mice analyzed at 0, 2, 4 and 8 weeks post-sham or LSI surgery. Scale bar, 1 mm. n = 8 per group. Data are shown as mean ± s.d. *p < 0.05, **p < 0.01.

Narrowed IVD space is often seen with human aging and regarded as a gold standard of DDD. Here, we employed a lumbar spine instability mouse model (LSI) (Fig. 1A) to examine whether altered mechanical loading in the spine leads to narrowing of IVD space. In detail, the L_3_–L_5_ spinous processes along with the supraspinous and interspinous ligaments were removed to induce instability of mouse lumbar spine (Fig. 1A). Mid-sagittal images of μCT suggested that the space between L_4_ and L_5_ vertebral bodies decreased in LSI mice, particularly in the posterior region (Fig. 1B). Analysis by three-dimensional reconstruction images of L_4–5_ EPs demonstrated that 8 weeks old mice continue to have growth of the spine, demonstrated by an increase in IVD volume 2 weeks post-sham surgery. The IVD volume then began to decrease up to 8 weeks post-sham surgery. In the LSI mice, there was no increase in IVD volume 2 weeks post-surgery, leading to statistically significant decrease in IVD volume relative to sham controls of similar age. The LSI mice continued to have a decreased IVD volume up to 8 weeks post-surgery relative to age matched sham controls (Fig. 1C,D).

### Narrowed IVD space is due to EP hypertrophy

**Figure 2:**
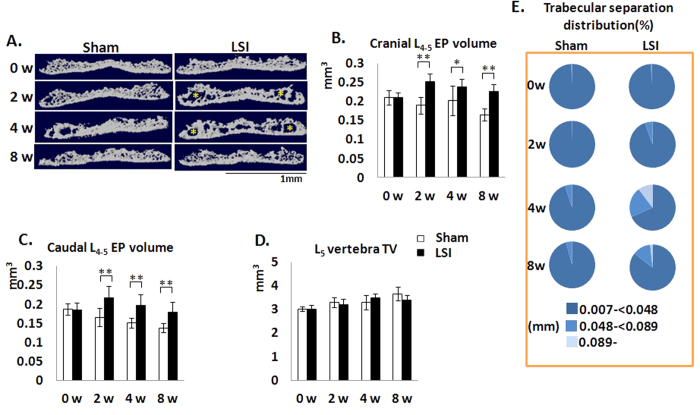
LSI leads to EP hypertrophy and increase in cavities. (**A**) Coronal plane of five consecutive images were used for 3D reconstruction of cranial EPs showing increased cavities in LSI mice, as indicated by asterisk. (**B**,**C**) Quantification of cranial (**B**) and caudal (**C**) EP volume obtained from μCT analysis. (**D**) Total tissue volume of L_5_ vertebrae as measured by μCT. (**E**) Trabecular separation was categorized by size presented as distribution percent of cavities in LSI EPs. Mice analyzed at 0, 2, 4 and 8 weeks post-sham or LSI surgery. Scale bar, 1 mm. n = 8 per group. Data are shown as mean ± s.d. *p < 0.05, **p < 0.01.

To explore if the narrowed IVD space in the unbalanced mechanical loading environments was caused by surrounding hard tissue enlargement, we examined the sizes of both cranial and caudal EPs as well as L_5_ vertebral body. The results showed that both cranial and caudal EP volumes were significantly increased post-surgery (Fig. 2B,C). An increased size of cavities within the EP were also noted in μCT 3-D images (Fig. 2A). The volume of L_5_ vertebral body was not affected (Fig. 2D). Further characterization of EP morphology by μCT analysis revealed an increase in trabecular separation distribution (Fig. 2E). Specifically, the percentage of values between 0.048 mm to 0.089 mm and above 0.089 mm reached to a peak at 4 w post-surgery, suggesting both the size and number of cavities were highest by this time point in LSI mice compared to the other time points in LSI mice (Fig. 2E right third). This value of EPs in 2 w-LSI mice was similar to that in 4 w-Sham mice, indicating LSI surgery accelerated the development of cavities in EPs. The percent of values greater than or equal to 0.089- were increased, but subsequently declined, suggesting it might be increased activity of EP remodeling in LSI mice.

### EP sclerosis is caused by accelerated EP bone remodeling

**Figure 3:**
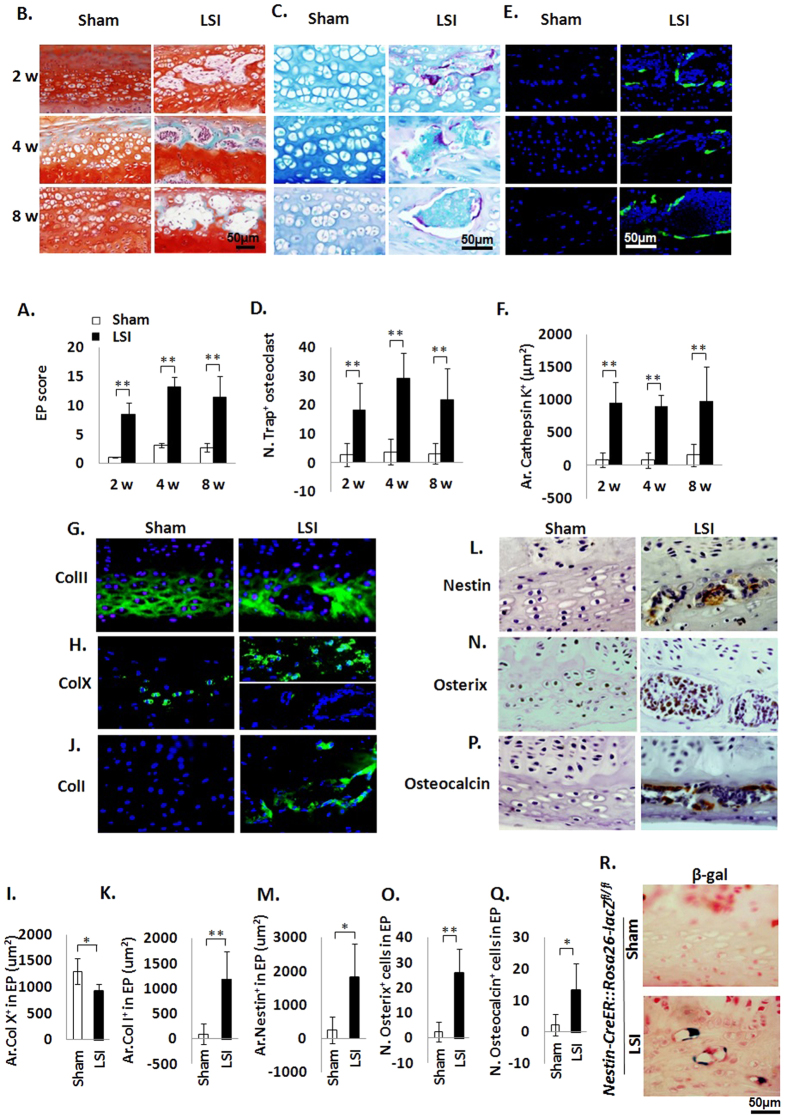
Accelerated bone remodeling in EPs of LSI mice. (**A**) EP score in LSI or sham mice as an indication of EP degeneration. **(B)** Representative images of Safranin O-Fast Green staining with cavities (green). (**C,D**) Representative Trap staining (purple) (**C**) and quantification (**D**). Methylene blue stains extracellular matrix (blue-green). (**E,F**) Representative images (**E**) and quantification (**F**) of immunofluorescence staining for Cathepsin K. DAPI stains nuclei blue. (**G,H,J**) Collagen II (**G**), Collagen X (**H**) and Collagen I (**J**) (green) immunofluorescence staining. DAPI stains nuclei blue. (**I**) Quantification in (**H**). (**K**) Quantification in (**J**). n = 6 per group. (**L**–**Q**) Representative staining (brown) and quantification of Nestin (**L,M**), Osterix (**N,O**), and Osteocalcin (**P**,**Q**) cells in EP cavities. Nuclei counterstained with hematoxylin (purple). n = 8 per group. (**R**) β-gal staining in *Nestin-CreER::Rosa26-lacZ*^*fl/fl*^mice with LSI operation *vs Nestin-CreER::Rosa26-lacZ*^*fl/fl*^mice with sham operation. n = 3 per group. Mice analyzed at 2, 4 and 8 weeks post-sham or LSI surgery (**A–F**) or 4 weeks (**G**–**R**) Scale bar, 50 μm. Data are shown as mean ± s.d. *p < 0.05, **p < 0.01.

To test if the change in EP cavity size was due to accelerated bone remodeling, we performed histological studies to examine how these cavities form. EP score, a histologic assessment of EP degeneration was applied for evaluation to account for pathological changes such as degrees of bony sclerosis, structure disorganization, and neovascularization^1^. We found EP score increased in the LSI mice compared to the Sham mice at similar time points, confirming EP degeneration (Fig. 3A). Safranin O and fast green staining suggested that EPs began to undergo endochondral ossification by 2 weeks post-surgery, as indicated by green-stained bone matrix surrounding the cavities in LSI mice relative to sham group (Fig. 3B). Interestingly, Trap and cathepsin K positive cells were noted beginning at 2 weeks post-surgery in LSI mice, whereas Trap and cathepsin K positive cells were rarely detected in sham controls at any time points (Fig. 3C–F), suggesting osteoclast resorptive activity increases after spinal instability.

The organization of type II collagen, which is normally detected in cartilage, was altered, with loss of type II collagen in the observed cavities and a change in spatial orientation in the remainder of the EP in LSI mice (Fig. 3G). Chondrocyte hypertrophy as assessed by type X collagen positive staining in LSI EPs decreased relative to the Sham EPs (Fig. 3I). Moreover, the distribution of the type X collagen positive cells was altered. In the Sham EPs, type X collagen positive cells were uniformly distributed (Fig. 3H Left), whereas in the LSI EPs, the positively staining cells were noted at sites where the chondrocytes merged together (Fig. 3H Right upper), but no positive cells were noted in larger EP cavities (Fig. 3H Right lower). Furthermore, expression of type I collagen, which is normally detected in ossified bone, was increased around the cavities in LSI EPs (Fig. 3J,K), which were regions where type II collagen were no longer expressed (Fig. 3E).

We performed further studies to determine if osteoblasts lineage cells were also present in the endochondral ossfication. Immunostaining for nestin, osterix and osteocalcin, markers of mesenchymal stem cells, pre-osteoblasts and mature osteoblasts, respectively, demonstrated a similar pattern as Trap staining. Specifically, all nestin, osterix and osteocalcin positive cells were abundant in the LSI mice compared to rarely detected in the sham controls (Fig. 3L–Q). Moreover, *Nestin-Cre*^*TM*^*Er::Rosa26-lacZ*^*flox/flox*^ mice were used to monitor the change of numbers and location of MSC lineage cells in EP after LSI operation. β-galactosidase (β-gal) staining of *Nestin-Cre*^*TM*^*Er::Rosa26-lacZ*^*flox/flox*^mice revealed that β-gal^+^ MSC lineage cells were increased in EP of LSI-operated mice, while no β-gal^+^ staining could be seen in EP of sham-operated mice (Fig. 3R).

### Upregulation of active TGFβ leads to EP degeneration and narrowed IVD space

**Figure 4:**
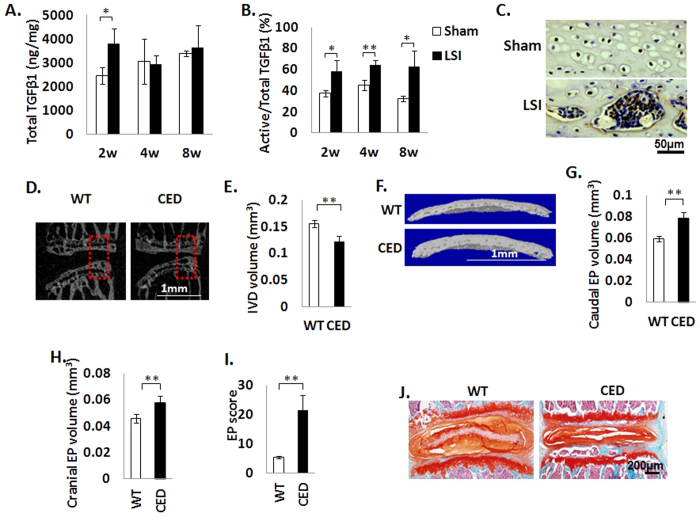
Upregulation of active TGFβ leads to EP hypertrophy and narrowed IVD space. (**A,B**) ELISA assay showing total TGFβ (**A**) or percent of active versus total TGFβ (**B**) in IVD at 2, 4 and 8 weeks post-sham or LSI surgery. n = 3 per group. (**C**) Immunostaining for pSmad2/3. Nuclei counterstained with hematoxylin (purple). (**D**) Mid-sagittal 3-D images of L_4_–L_5_ of CED and WT mice. Scale bar, 1 mm. Red rectangle represents the region of interest (ROI) chosen for calculation of IVD volume shown in (**E**). (**F**) μCT 3-D images of cranial EP of CED mice and their WT littermates. Scale bar, 1 mm. (**G,H**) Quantitative analysis of ROI of cranial (**G**) and caudal (**H**) EP volume. (**I**) EP score of CED mice and their WT littermates as an indication of EP degeneration. (**J**) Safranin O-Fast Green staining of IVD sections showing increased ossification (green) of EP and decrease of IVD height in CED mice. Scale bar, 200 μm. n = 6 per group. Data are shown as mean ± s.d. *p < 0.05, **p < 0.01.

Excess activation of TGFβ has been found to contribute to the pathogenesis of sclerotic subchondral bone leading to OA^7^,^8^. In our LSI mouse model, the total TGFβ1 protein level was elevated 2 weeks post-LSI surgery, but was not significantly changed at later time points (Fig. 4A). The percentage of TGFβ that was active was increased at 2, 4 and 8 weeks post-LSI surgery (Fig. 4B). Downstream TGFβ signaling was confirmed by IHC examination, showing pSmad2/3 positive staining in both hypertrophic chondrocytes and bone marrow cells in EP cavities (Fig. 4C). To determine if the elevated TGFβ was involved in EP degeneration, we utilized a transgenic mouse model (CED) that results in overexpression of active TGFβ driven by a 2.3-kb type I collagen promoter^13^, as type I collagen is mainly expressed in the outer layer of the annulus fibrosis and is in direct contact with the EPs^15^. The EP phenotype in the CED mice was similar to the LSI mice. Specifically, mid-sagittal μCT scan revealed that the IVD space between L_4_ and L_5_ vertebral bodies significantly narrowed in CED mice relative to their WT littermates (Fig. 4D,E). Three-dimensional reconstruction and analysis of L_4–5_ EPs revealed a significant increase in both cranial and caudal EP volumes in CED versus WT littermates (Fig. 4F–H). Increased EP score was observed in CED mice compared to WT littermates (Fig. 4I). Histologic analysis confirmed that the decreased IVD height was associated with endochondral ossification of EPs in CED mice relative to their WT littermates (Fig. 4J). Taken together, the data indicate that upregulation of TGFβ is involved in sclerosis and hypertrophy of EP, which occurred in association with narrowing of IVD space.

### Inhibition of TGFβ signaling attenuates EP degeneration in LSI mice

**Figure 5:**
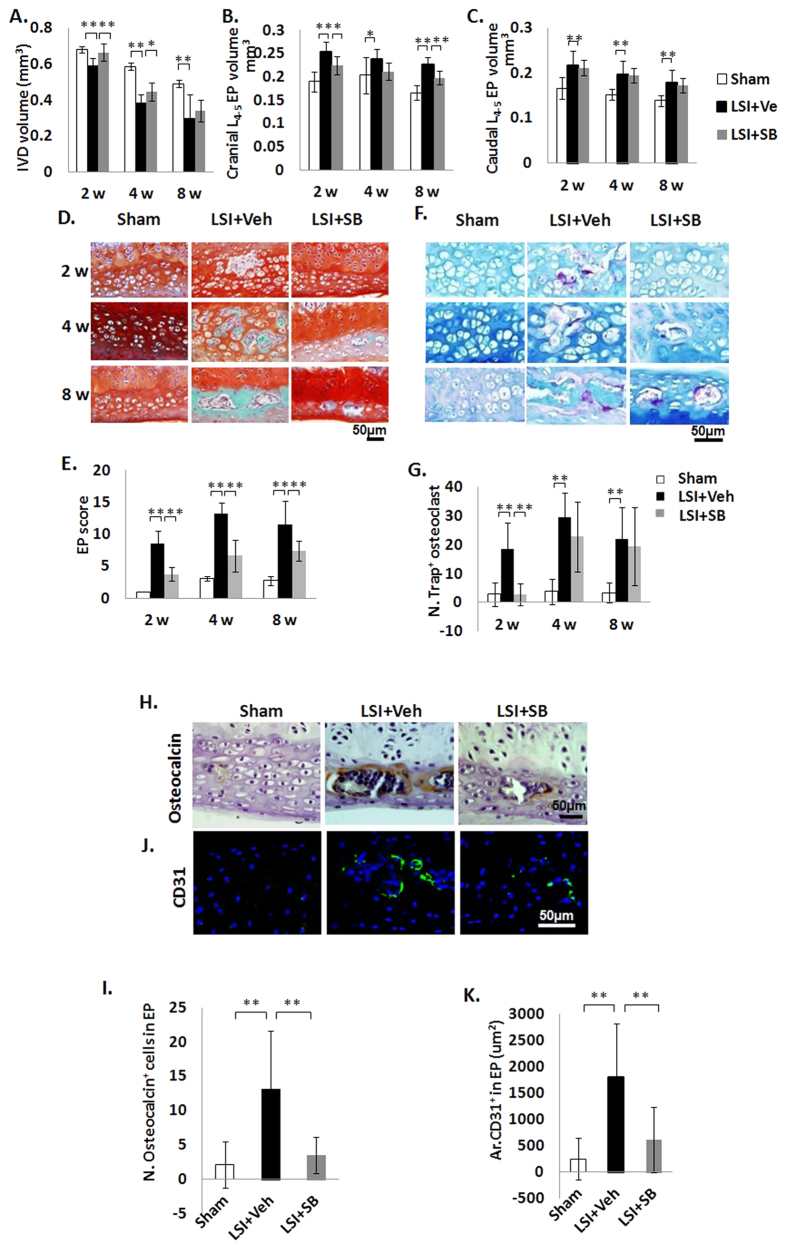
Inhibition of excess active TGFβ attenuates EP degeneration in LSI mice. (**A**–**C**) IVD volume (**A**), Cranial (**B**) and Caudal (**C**) EP volume by μCT analysis. (**D**) Safranin O and fast Green staining in Sham or LSI mice treated with vehicle or LSI mice treated with TβRI inhibitor (SB). (**E**) EP score as an indication of EP degeneration. (**F,G**) Trap staining (purple) (**F**) and Quantitative analysis (**G**). Methylenebluestains extracellular matrix (blue-green). (**H**) Immunostaining for osteocalcin (brown). Hematoxylin stains nuclei purple. (**I**) Quantification of (**H**) (**J**,**K**) Immunofluorescence staining (**J**) and quantification for CD31 (green) (**K**). DAPI stains nuclei blue. Mice analyzed at 2, 4 and 8 weeks post-sham or LSI surgery (**A–G**) or 4 weeks (**H–K**). n = 8 per group. Scale bar, 50 μm. Data are shown as mean ± s.d. *p < 0.05, **p < 0.01.

We then examined if EP degeneration could be prevented by inhibiting TGFβ signaling. TGFβ type I receptor (TβRI) inhibitor (SB, 1 mg/kg) was systemically injected in the LSI mice. Lumbar spine samples were collected at 2, 4 and 8 w post-surgery. We found IVD volume increased after 2 and 4 weeks treatment of inhibitor in LSI mice relative to LSI treated with vehicle (Fig. 5A). Cranial EP volume was preserved with inhibitor treatment as indicated by no statistically significant difference relative to sham controls (Fig. 5B). However, no effect was were observed in caudal EP volume, as EP volumes in LSI SB groups were similar to LSI sham controls (Fig. 5C). The ossification and degeneration of EPs were improved, although not completely abrogated by TβRI inhibitor as indicated by EP scores intermediate between sham and LSI treated with vehicle controls (Fig. 5D,E). Osteoclast numbers were decreased to sham controls levels in the LSI TβRI inhibitor treated mice, but this effect was not sustained at later time points (Fig. 5F,G). On another hand, the number of osteocalcin^+^ cells was decreased in LSI mice by inhibitor treatment for 4 weeks (Fig. 5H,I). Osteogenesis is coupled with angiogenesis^16^. Therefore, we also evaluated if the blood vessel was affected by TGFβ inhibition. We found CD31^+^ staining was increased after LSI operation, but was preserved by inhibitor treatment, indicating the inhibitory effect of TβRI inhibitor in angiogenesis (Fig. 5J,K).

### Inhibition of excess activation of TGFβ attenuates EP degeneration in CSI mice

**Figure 6:**
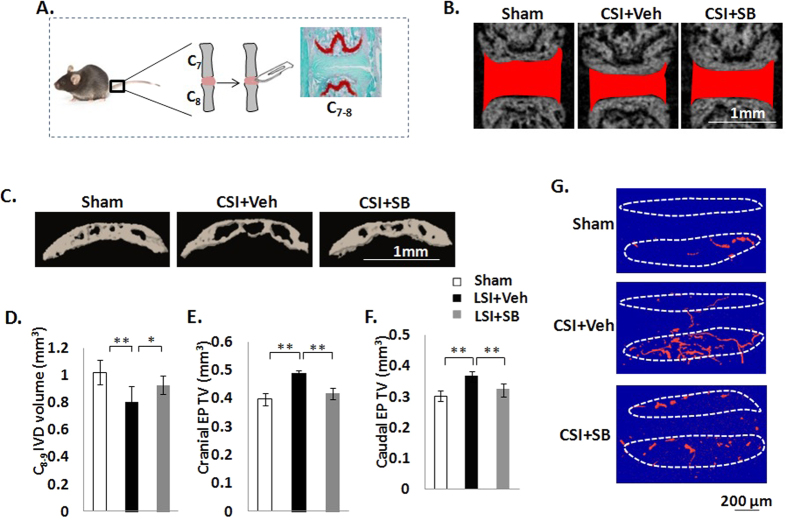
Inhibition of excess active TGFβ attenuates EP degeneration in CSI mice. (**A**) Caudal spine instability mouse model (CSI). The instability of caudal spine was induced by full depth annular stab and NP removal of the C_7–8_ IVD. CSI or Shan mice were treated with either vehicle (Veh) or TβRI inhibitor (SB) as indicated. C_7–8_ EPs were analyzed. (**B**) Coronal plane of C_7–8_ IVD space indicated by red color. Scale bar, 1 mm. (**C**) Coronal plane of five consecutive images were used for 3D reconstruction of caudal EPs. Scale bar, 1 mm. (**D–F**) Quantification of IVD volume, cranial (**E**) and caudal (**F**) EP volume by μCT analysis. (**G**) 3D-images of CT-based microangiography. Scale bar 200 μm. n = 6 per group. Data are shown as mean ± s.d. **p < 0.01, *p < 0.05.

To further evaluate the effect of TGFβ inhibitor in EP degeneration, we employed second spinal instability model, CSI and injected with either vehicle or TβRI inhibitor (Fig. 6A). In this model, caudal 7^th^–8^th^ (C_7–8_) IVD was injured by annular stab and NP removal to induce caudal spine instability. IVD and EP volumes were preserved with similar volumes to sham controls when LSI mice were treated with TβRI inhibitor (Fig. 6B–E). We also evaluated the effect of TGFβ inhibitor on the blood vessel volume. After injury, more blood vessels could be seen in both cranial and caudal EPs of CSI mice whereas they were decreased after TβRI inhibitor treatment (Fig. 6F).

## Discussion

EPs function as transitional tissue that absorbs hydrostatic pressure resulting from mechanical loading of the spine. In our previous study, EP hypertrophy was found in rat lumbar IVD when the axial force was increased upon the spine^9^. Cadaveric lumbar spines also show a similar trend of more severe IVD degeneration associated with greater thickness in both the cranial and caudal EPs^17^. In this study, we revealed that unstable mechanical loading in the spine induces EPs hypertrophy and is associated with IVD degeneration. We systematically analyzed the changes of cartilaginous EP, IVD space and vertebral bodies in the spine in two different spine instability animal models, considering them as a functional unit. The responses of EP resulted in narrowing of the IVD space and likely generated pathological static compression on the IVDs.

In pathological conditions, static compression stress activates excess TGFβ^18^,^19^. In the physiologic state, TGFβ maintains chondrocyte homeostasis to preserve EP structure and function^20^,^21^,^22^. In our current study, we found that elevated TGFβ was associated with accelerated endochondral ossification and vascularization in EP regions as indicated by hypertrophic chondrocytes activity and the presence of osteoclasts, osteoblasts, endothelial cells in LSI mouse model. These changes were observed without spinal instability surgery in the transgenic mice with overexpression of TGFβ and attenuated when TGFβ signaling was inhibited in the LSI mice. These changes are similar to those found in subchondral bone of OA patients and animal models^7^. TGFβ released from latent extracellular matrix mobilizes and recruits MSCs^23^,^24^,^25^. MSCs are thought to localize with vasculature^26^. We found that endothelial cells and osteoblast lineage cells appeared at a similar time in EPs to support osteogenesis. As TGFβ signaling exerts its primary effect on MSC migration, rather than osteoclast activity^25^, we did not, as expected, see effect on osteoclast numbers when TGFβ signaling was inhibited. The temporary early decrease in osteoclast numbers observed in the LSI + SB group may have been due inhibition of immunological reactions associated with osteoclast function^27^.

EP degeneration is thought to begin with abnormal calcification^28^, a process where calcium crystals salts are deposited into pores of EP. The calcified EP undergoes ossification and are eventually replaced with bone during aging^6^,^17^. The process is thought to reduce nutrients transport from vertebral marrow to IVD^6^. In our study, we found spinal instability accelerated ossification of EPs in the LSI mouse model and contributed to EP hypertrophy and sclerosis. The CSI mouse model was utilized as a second spinal instability model. The caudal spine in mice, unlike the lumbar spine, undergoes spontaneous ossification at a younger age with complete ossification at the 8 week age studied^29^. Similar to the LSI model, the CSI model also demonstrated EP hypertrophy likely from abnormal bone remodeling in the osseous EP area. Although bone remodeling is coupled with angiogenesis and reduces the distance between circulating nutrients and IVD^30^,^31^, the nutrient diffusion is actually impaired as a consequence of the replacement with cortical bone matrix^32^. The cortical bone matrix type I collagen in conjunction with loss of local type II collagen and change in spatial orientation contributes to EP sclerosis.

TGFβ has been found to increase proteoglycan expression and exert an anabolic effect to alter IVD development and degeneration^20^,^22^,^33^,^34^,^35^. However our study suggests that supraphysiologic levels of TGFβ can also be detrimental to the IVD. Further supporting this idea are reports that high levels of TGFβ1 are present in the IVDs from DDD patients^36^,^37^,^38^,^39^ and a rabbit model of IVD benefit with TGFβ inhibition^33^. It is most likely that TGFβ has a functional versatility on the metabolism of IVD cells, where both too little and excessive signaling are detrimental.

We found that alterations in mechanical loading increase TGFβ early in the course of DDD. TGFβ is associated with ossification of the EP, leading to EP hypertrophy and likely static compression of the IVD by narrowing IVD space. Inhibition of TGFβ activation in the early phase of DDD attenuated EP and IVD degeneration.

## Additional Information

**How to cite this article**: Bian, Q. *et al.* Excessive Activation of TGFβ by Spinal Instability Causes Vertebral Endplate Sclerosis. *Sci. Rep.* **6**, 27093; doi: 10.1038/srep27093 (2016).
